# Surgery-enabled precision oncology in an MSI-High pulmonary artery sarcoma with Lynch syndrome: a case report

**DOI:** 10.3389/fonc.2026.1822606

**Published:** 2026-04-30

**Authors:** Akina Nigi, Keisuke Iwamoto, Hidetoshi Itani, Shigeto Kondou, Junzi Uraki, Toshiya Tokui

**Affiliations:** 1 Department of Respiratory Medicine, Japanese Red Cross Ise Hospital, Ise, Japan; 2 Department of Radiology, Japanese Red Cross Ise Hospital, Ise, Japan; 3 Department of Thoracic Surgery, Japanese Red Cross Ise Hospital, Ise, Japan

**Keywords:** immune checkpoint inhibitor, Lynch syndrome, microsatellite instability-high (MSI-H), pulmonary artery sarcoma, tumor mutation burden

## Abstract

This report describes a rare case of a vascular-origin malignant tumor (intimal sarcoma or carcinosarcoma) extending from the right pulmonary hilum along the pulmonary artery in a 44-year-old man. The patient underwent right pneumonectomy under extracorporeal membrane oxygenation (ECMO) support and achieved long-term survival through molecularly guided immunotherapy. His medical history included two early-onset colorectal cancers and a family history of malignancy, suggesting an underlying hereditary cancer syndrome. The tumor exhibited microsatellite instability–high (MSI-high) status and a high tumor mutational burden (TMB-high) with low PD-L1 expression but responded favorably to immune checkpoint inhibitors, including ipilimumab plus nivolumab and pembrolizumab, achieving sustained disease stability for over 65 months. Comprehensive genomic profiling conducted as part of a branch study later in the disease course revealed a pathogenic germline MLH1 variant, confirming the diagnosis of Lynch syndrome. This case underscores the importance of surgery-enabled precision oncology in the management of rare and aggressive tumors.

## Introduction

Pulmonary artery sarcomas are exceedingly rare and aggressive intravascular malignancies that often masquerade as pulmonary thromboembolism, delaying diagnosis and leading to poor outcomes. These tumors lack standardized systemic therapy, and prognosis remains dismal despite advances in surgical techniques and multimodal treatment approaches.

Immune checkpoint inhibitors (ICIs) have revolutionized cancer therapy, yet their efficacy in soft tissue sarcomas has been modest and largely limited to molecularly defined subtypes. Predictive biomarkers such as microsatellite instability-high (MSI-high) and high tumor mutational burden (TMB) are known to correlate with ICI responsiveness in several tumor types, but their relevance in vascular-origin sarcomas remains largely unestablished.

Here, we describe a patient with a rapidly progressive pulmonary artery carcinosarcoma that was managed with life-saving pneumonectomy under extracorporeal membrane oxygenation (ECMO) support. Surgical resection enabled comprehensive molecular profiling, which revealed MSI-high and TMB-high status, ultimately leading to biomarker-driven ICI therapy and exceptional long-term disease control.

Notably, germline testing, BRANCH study, confirmed a pathogenic MLH1 mutation, establishing a diagnosis of Lynch syndrome. While Lynch syndrome is classically associated with colorectal, endometrial, and other epithelial malignancies, its link to sarcomas is exceedingly rare and controversial.

This case underscores the importance of surgery as an enabler of precision oncology in aggressive and diagnostically challenging tumors. It highlights how rare tumors, when viewed through the lens of molecular oncology, can defy conventional therapeutic expectations.

## Case presentation

**Figure 1:**
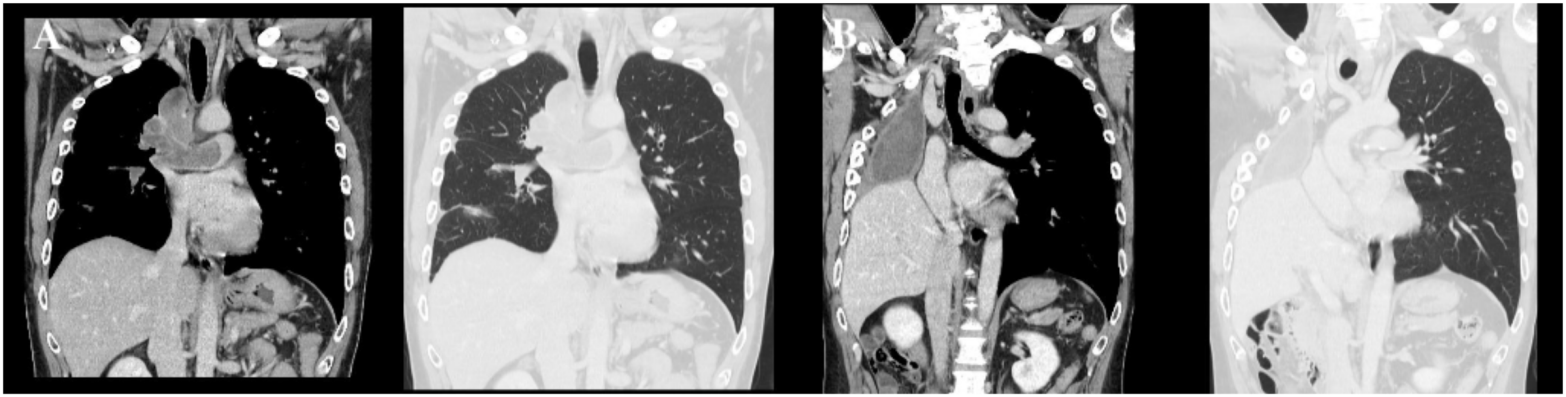
**(A)** Preoperative contrast-enhanced CT in the coronal plane showing an intravascular tumor extending within the pulmonary artery. **(B)** Postoperative contrast-enhanced CT in the same coronal plane demonstrating disappearance of the intravascular lesion after surgical resection.

A 44-year-old man presented with acute chest pain and was referred for further evaluation after imaging revealed abnormal findings in the right pulmonary hilum. Contrast-enhanced computed tomography demonstrated a large intraluminal mass occupying the right main pulmonary artery with extension into the lobar pulmonary arteries, forming a cast-like structure within the vascular lumen (**Figure 1A**). No distinct pulmonary parenchymal mass was identified. Peripheral pulmonary infarction was also observed, raising concern for imminent vascular occlusion and sudden death.

Given the life-threatening nature of the lesion and the high risk of catastrophic pulmonary artery obstruction, systemic therapy or radiotherapy was deemed unsafe as an initial approach. The patient therefore underwent emergency right pneumonectomy with pulmonary artery resection under ECMO support. Complete macroscopic resection was achieved (**Figure 1B**).

**Figure 2:**
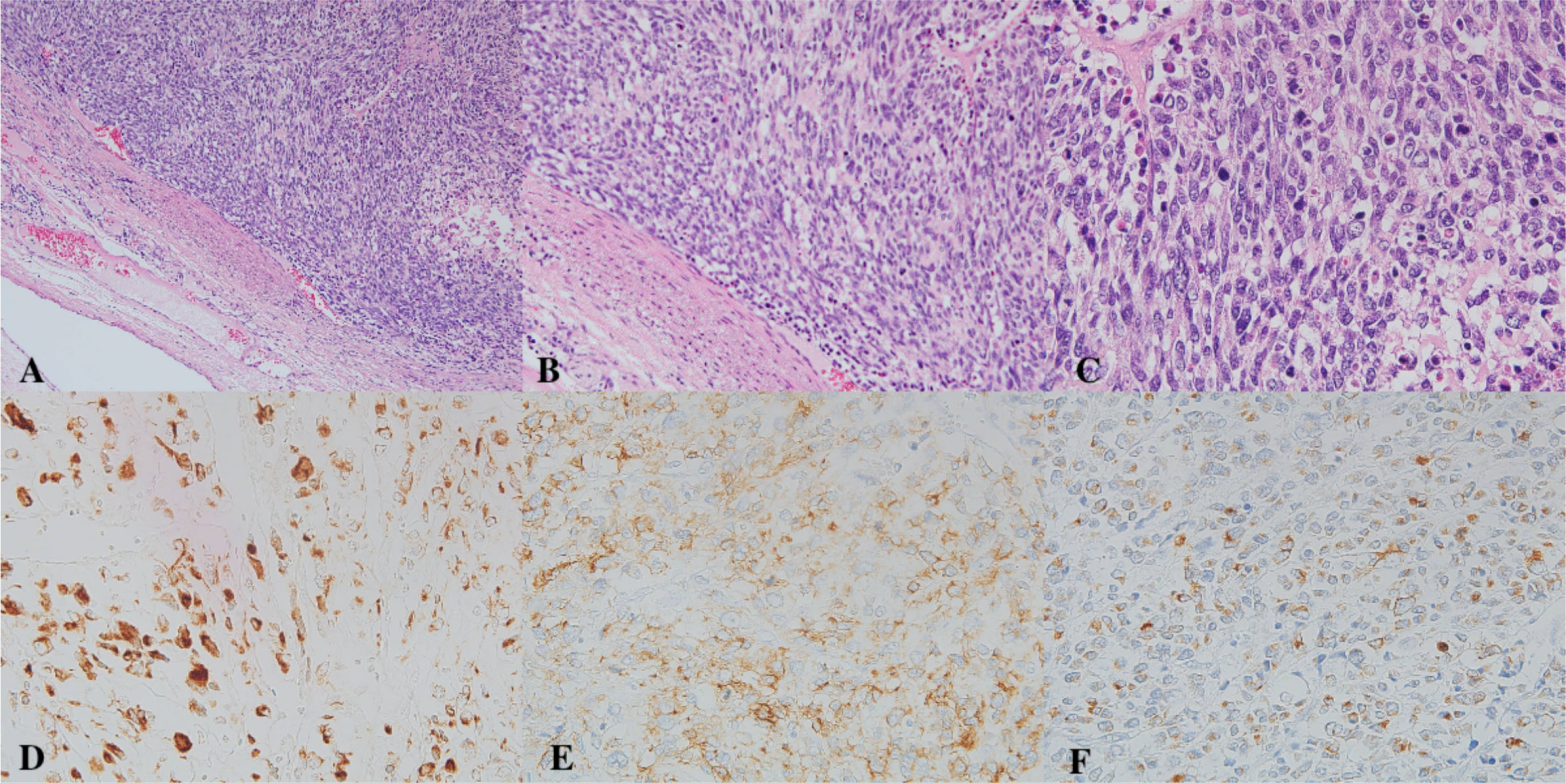
Histopathological and immunohistochemical findings of the pulmonary artery tumor. **(A)** Hematoxylin and eosin (H&E) staining at ×100 magnification shows spindle cell proliferation within and along the vascular wall, adjacent to vascular smooth muscle. **(B)** H&E staining at ×200 magnification demonstrates infiltration of the spindle cell tumor into the vascular smooth muscle. **(C)** H&E staining at ×400 magnification reveals marked cytologic atypia with scattered mitotic figures. **(D)** Immunohistochemistry at ×400 magnification shows focal cytokeratin positivity in pleomorphic, polygonal, undifferentiated tumor cells. **(E)** Immunohistochemistry at ×400 magnification demonstrates α-smooth muscle actin (α-SMA) positivity in the same area as panel **(D, F)** Immunohistochemistry at ×400 magnification shows negative staining for desmin.

Histopathological examination revealed a malignant tumor composed predominantly of spindle and pleomorphic cells with focal cartilaginous differentiation. Immunohistochemistry demonstrated focal positivity for cytokeratin AE1/AE3 and mesenchymal markers, while endothelial markers were negative. The overall findings were consistent with a diagnosis of pulmonary artery carcinosarcoma or intimal sarcoma (**Figure 2**).

Postoperatively, the patient recovered without major complications. Eight months after surgery, surveillance imaging identified a solitary recurrence in the left adrenal/renal region (**Supplementary Figure 1**). Given the absence of other metastatic lesions and the patient’s prior clinical course, systemic therapy was initiated.

## Molecular findings

Comprehensive genomic profiling of the resected tumor was performed using next-generation sequencing. The tumor demonstrated MSI-high status and a high tumor mutational burden of 16 mutations per mega-base. Pathogenic alterations in DNA damage response and chromatin remodeling genes were identified, including frameshift and nonsense mutations in TP53, as well as mutations in ATRX, SETD2, KMT2D/MLL2, EDD, HDAC1 (**Supplementary Table 1**).

## Treatment and outcome

Following detection of postoperative recurrence, the patient was treated with immune checkpoint inhibition using combination ipilimumab and nivolumab. Because pleural thickening was initially suspected to represent tumor progression, docetaxel plus ramucirumab was administrated. However, subsequent evaluation revealed that pleural thickening was reactive rather than malignant, and treatment was therefore switched to pembrolizumab. Treatment was generally well tolerated, with manageable immune-related adverse events. A family history suggested hereditary tumors, but testing was not performed at the patient’s request. After the relationship between the patient and attending physician was noted, testing was pursued again and consent was obtained.

A pathogenic germline variant in MLH1 was identified, confirming the diagnosis of Lynch syndrome. This finding was supported by the patient’s medical history of two early-onset colorectal cancers and a family history of malignancies, as summarized in the pedigree (**Supplementary Table 2**).

Radiographic evaluation demonstrated disease stabilization after initiation of immunotherapy. Notably, durable disease control has been maintained for 65 months following the start of immune checkpoint inhibition, with no evidence of progressive disease. The patient has remained functionally independent and continues full-time employment.

This prolonged disease stability far exceeds the expected clinical course for pulmonary artery sarcoma and represents an exceptional response to immunotherapy in this disease context. The chronological timeline of the case presentation is summarized in **Table 1**.

**Table 1:** Timeline of the patient’s clinical course and treatments.

Month 1	Initial presentation with acute chest pain.
Month 1	Referred to our hospital; contrast-enhanced computed tomography revealed a large intraluminal tumor in the right pulmonary artery.
Month 2	Emergency surgery under extracorporeal membrane oxygenation (ECMO): right pneumonectomy with lymph node dissection.
Month 5	Postoperative recurrence detected in the left adrenal gland.
Month 7	Initiation of immune checkpoint inhibition with ipilimumab plus nivolumab.
Month 21	Suspected pleural recurrence with pleural thickening. Sequential chemotherapy administered (docetaxel + ramucirumab, followed by FN, then carboplatin + pemetrexed). Subsequent evaluation suggested reactive pleural thickening rather than true disease progression.
Month 27	Pembrolizumab initiated and continued as maintenance therapy, achieving sustained stable disease.
Month 65	At 65 months after initiation of immune checkpoint inhibition, the patient remains alive with ongoing disease control, has fully returned to social and occupational activities, and continues active treatment.

## Discussion

Pulmonary artery intravascular sarcomatous tumors, including intimal sarcoma–like tumors, angiosarcoma, and carcinosarcoma-like variants, are extremely rare and aggressive malignancies with a dismal prognosis. Although sporadic case reports have described pulmonary artery sarcomas or vascular sarcomas, long-term survival remains exceptional, and no established systemic therapy exists for recurrent disease (1–6).

A potential association between pulmonary artery sarcoma and Lynch syndrome has been previously suggested; however, genetic confirmation has been limited. Notably, the only prior report describing a pulmonary artery intimal sarcoma in the context of suspected Lynch syndrome was based on a variant of uncertain significance in PMS2, without definitive germline confirmation. In that case, immune checkpoint inhibition achieved transient disease control for approximately 17 months (7, 8).

In contrast, the present case is distinguished by genetically confirmed Lynch syndrome, demonstrated by a pathogenic germline variant in MLH1, established through comprehensive germline testing and genetic counseling. The tumor exhibited mismatch repair deficiency and microsatellite instability–high status, providing a strong biological rationale for immune checkpoint inhibition (9–11). In addition, recent clinical evidence, including CheckMate 8HW, has further supported the activity of combined immune checkpoint blockade in dMMR/MSI-high malignancies, although the available data are derived largely from colorectal cancer rather than sarcoma (12).

Importantly, immune checkpoint inhibition resulted in durable disease control for approximately 65 months after recurrence, far exceeding outcomes reported in previous cases (13–15).

**Figure 3:**
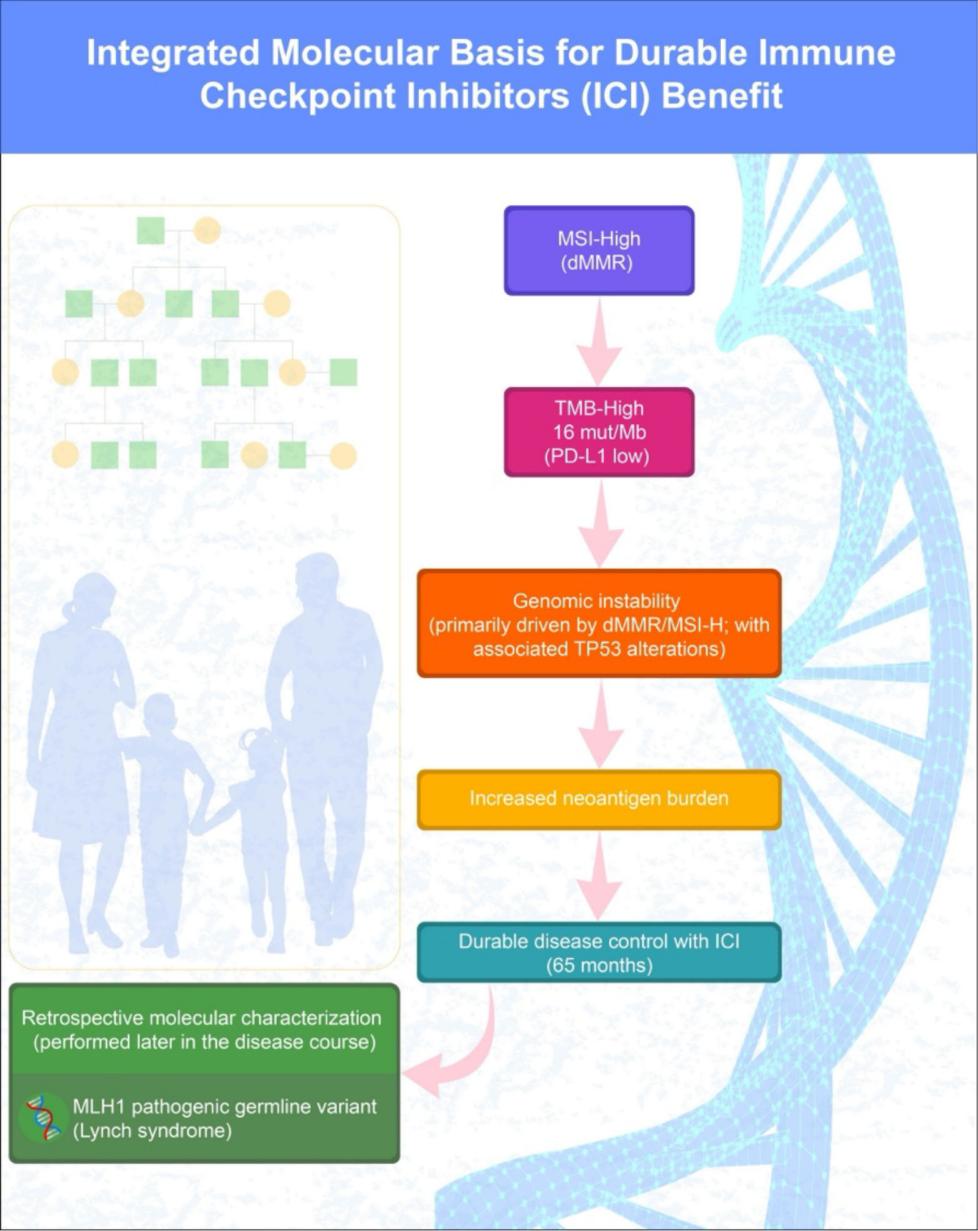
Integrated molecular basis for durable response to immune checkpoint inhibition. A high level of microsatellite instability (MSI-H) and tumor mutational burden (TMB-H), despite low PD-L1 expression, suggested potential responsiveness to immunotherapy. Underlying genomic instability, including TP53 and chromatin remodeling gene alterations, contributed to increased neoantigen load. Retrospective germline analysis revealed a pathogenic MLH1 mutation, confirming Lynch syndrome. These converging features underpinned durable disease control lasting 65 months.

The exceptional durability of response observed in this case is likely attributable to several converging biological and clinical factors. These converging biological factors and their relationship to durable immune checkpoint inhibitor efficacy are summarized in **Figure 3**.

In this model, mismatch repair deficiency driven by a pathogenic germline MLH1 variant resulted in microsatellite instability–high status, leading to an elevated tumor mutational burden despite low PD-L1 expression.

Accumulated genomic instability, including TP53 and chromatin remodeling gene alterations, likely increased neoantigen burden and intrinsic tumor immunogenicity, ultimately enabling durable immune-mediated disease control.

First, the presence of a pathogenic germline MLH1 variant provides a robust biological basis for immune checkpoint inhibition in an MSI-high/dMMR tumor (9–11). We speculate that durable mismatch repair deficiency and a persistently elevated neoantigen burden may have contributed to the unusually prolonged response observed in this case.

Second, multiple co-occurring alterations affecting genomic stability and chromatin regulation—including TP53, MSH6, ATRX, SETD2, KMT2D (MLL2), EED, and HDAC1—may have shaped the tumor’s immunologic landscape. Although the functional contribution of each alteration cannot be determined, such genomic complexity has been associated with enhanced tumor immunogenicity in selected contexts (13–18). Notably, despite low PD-L1 expression, immune checkpoint inhibition achieved durable disease control, suggesting that intrinsic tumor immunogenicity rather than PD-L1–mediated adaptive immune resistance may have been the dominant driver of response (13–18).

In addition, surgery played a critical role not only as a life-saving intervention but also as an enabler of precision oncology. Without emergency surgical resection under extracorporeal membrane oxygenation support, the patient would not have survived long enough to undergo comprehensive molecular profiling or receive biomarker-guided systemic therapy (3–5). Moreover, surgical intervention enabled accurate histopathologic assessment and molecular characterization, which were essential for subsequent biomarker-driven treatment decisions.

Although the histopathologic classification of this tumor does not fit neatly into a single category—given its intravascular growth pattern, sarcomatous morphology, focal epithelial differentiation, and absence of MDM2 amplification—it most appropriately falls within the spectrum of high-grade intravascular sarcomatous tumors of the pulmonary artery (6). Recent genomic profiling studies have demonstrated that intimal sarcomas comprise distinct molecular subtypes with heterogeneous tumor microenvironments, underscoring the biological diversity of this entity (19–21). This diagnostic ambiguity highlights a central message of this case: in rare aggressive tumors, molecular features may be more informative than histologic labels alone for therapeutic decision-making (7, 8).

During the disease course, a solitary adrenal lesion developed in the absence of other metastatic lesions. Although histopathologic confirmation was not obtained, the temporal relationship and complete radiologic resolution strongly support metastatic disease rather than a benign adrenal lesion, further reinforcing the systemic antitumor activity of immunotherapy in this case (3–5).

It is important to emphasize that mismatch repair deficiency and Lynch syndrome should not be generalized as predictors of immune checkpoint inhibitor efficacy across all tumor types. While mismatch repair deficiency provides a biological rationale for immunotherapy, durable responses remain uncommon, particularly in sarcomatous and vascular tumors. Accordingly, this case should be interpreted not as evidence of universal efficacy, but rather as an exceptional responder illustrating the potential value of integrating germline genetics, tumor molecular profiling, and careful clinical judgment (16–18).

## Conclusion

This case represents, to our knowledge, the first report of a genetically confirmed Lynch syndrome–associated pulmonary artery intravascular sarcomatous tumor achieving durable disease control with immune checkpoint inhibition. These findings highlight the importance of a surgery-enabled, biomarker-driven precision oncology approach in rare and aggressive malignancies for which standard therapies are lacking.

## Data Availability

The original contributions presented in this study are included in the article and its **Supplementary Material**. Further inquiries can be directed to the corresponding author.

## References

[1] Qin J Ng CS He P Lin X Lin X Hou P . Pulmonary artery intimal sarcoma - a primeval or rediscovered tumor? A report of 14 new cases with literature review. Pathol Res Pract. (2021) 224:153548. doi: 10.1016/j.prp.2021.153548. PMID: 34280751

[2] Zhao M Nie P Guo Y Chen H . Pulmonary artery intimal sarcoma: a rare cause of filling defects in pulmonary arteries. Am J Med Sci. (2022) 364:655–60. doi: 10.1016/j.amjms.2022.05.009. PMID: 35588894

[3] Han Y Zhen Y Liu X Zheng X Zhang J Zhai Z . Surgical treatment of primary pulmonary artery sarcoma. Gen Thorac Cardiovasc Surg. (2021) 69:638–45. doi: 10.1007/s11748-020-01476-2. PMID: 32918676 PMC7981312

[4] Blackmon SH Rice DC Correa AM Mehran R Putnam JB Smythe WR . Management of primary pulmonary artery sarcomas. Ann Thorac Surg. (2009) 87:977–84. doi: 10.1016/j.athoracsur.2008.08.018. PMID: 19231448

[5] Yin K Zhang Z Luo R Ji Y Zheng D Lin Y . Clinical features and surgical outcomes of pulmonary artery sarcoma. J Thorac Cardiovasc Surg. (2018) 155:1109–1115.e1. doi: 10.1016/j.jtcvs.2017.10.101. PMID: 29223846

[6] Xu L Lu W Li J Wang C . Additional treatment prolonged survival of pulmonary artery sarcoma after surgical resection. Transl Cancer Res. (2020) 9:2618–26. doi: 10.21037/tcr.2020.02.80. PMID: 35117621 PMC8799134

[7] Mounai Y Yoshida T Ito S Fukuda K Shimazu K Taguchi D . Pulmonary artery intimal sarcoma in a patient with Lynch syndrome: response to an immune checkpoint inhibitor. Case Rep Oncol. (2023) 16:21–9. doi: 10.1159/000528682. PMID: 36743879 PMC9891846

[8] Poumeaud F Valentin T Vande Perre P Jaffrelot M Bonnet D Chibon F . Special features of sarcomas developed in patients with Lynch syndrome: a systematic review. Crit Rev Oncol Hematol. (2023) 188:104055. doi: 10.1016/j.critrevonc.2023.104055. PMID: 37301271

[9] Boyiadzis MM Kirkwood JM Marshall JL Pritchard CC Azad NS Gulley JL . Significance and implications of FDA approval of pembrolizumab for biomarker-defined disease. J Immunother Cancer. (2018) 6:35. doi: 10.1186/s40425-018-0342-x. PMID: 29754585 PMC5950135

[10] Marcus L Lemery SJ Keegan P Pazdur R . FDA approval summary: pembrolizumab for the treatment of microsatellite instability-high solid tumors. Clin Cancer Res. (2019) 25:3753–8. doi: 10.1158/1078-0432.CCR-18-4070. PMID: 30787022

[11] First tissue-agnostic drug approval issued. Cancer Discov. (2017) 7:656. doi: 10.1158/2159-8290.CD-NB2017-078. PMID: 28583911

[12] Andre T Elez E Van Cutsem E Jensen LH Bennouna J Mendez G . Nivolumab plus ipilimumab in microsatellite-instability-high metastatic colorectal cancer. N Engl J Med. (2024) 391:2014–26. doi: 10.1056/NEJMoa2402141. PMID: 39602630

[13] Tawbi HA Burgess M Bolejack V Van Tine BA Schuetze SM Hu J . Pembrolizumab in advanced soft-tissue sarcoma and bone sarcoma (SARC028): a multicentre, two-cohort, single-arm, open-label, phase 2 trial. Lancet Oncol. (2017) 18:1493–501. doi: 10.1016/S1470-2045(17)30624-1. PMID: 28988646 PMC7939029

[14] D'Angelo SP Mahoney MR Van Tine BA Atkins J Milhem MM Jahagirdar BN . Nivolumab with or without ipilimumab treatment for metastatic sarcoma (Alliance A091401): two open-label, non-comparative, randomised, phase 2 trials. Lancet Oncol. (2018) 19:416–26. doi: 10.1016/S1470-2045(18)30006-8. PMID: 29370992 PMC6126546

[15] Wagner MJ Othus M Patel SP Ryan C Sangal A Powers B . Multicenter phase II trial (SWOG S1609, cohort 51) of ipilimumab and nivolumab in metastatic or unresectable angiosarcoma: a substudy of dual anti-CTLA-4 and anti-PD-1 blockade in rare tumors (DART). J Immunother Cancer. (2021) 9:e002990. doi: 10.1136/jitc-2021-002990. PMID: 34380663 PMC8330584

[16] Fernandes I Dias E Silva D Segatelli V Filippi RZ Carolina de Rezende A Campregher P . Microsatellite instability and clinical use in sarcomas: systematic review and illustrative case report. JCO Precis Oncol. (2024) 8:e2400047. doi: 10.1200/PO.24.00047. PMID: 39432881

[17] Denu RA Quintana-Perez CD Wangsiricharoen S Ingram DR Wani KM Lazar AJ . DNA mismatch repair deficiency as a biomarker in sarcoma. Surg Oncol Insight. (2024) 1:100091. doi: 10.1016/j.soi.2024.100091. PMID: 40190387 PMC11967435

[18] Wood GE Meyer C Petitprez F D'Angelo SP . Immunotherapy in sarcoma: current data and promising strategies. Am Soc Clin Oncol Educ Book. (2024) 44:e432234. doi: 10.1200/EDBK_432234. PMID: 38781557

[19] WHO Classification of Tumours Editorial Board . Soft tissue and bone tumours. In: WHO classification of tumours series, 5th ed, vol. 3. International Agency for Research on Cancer, Lyon (France (2020).

[20] Koelsche C Benhamida JK Kommoss FKF Stichel D Jones DTW Pfister SM . Intimal sarcomas and undifferentiated cardiac sarcomas carry mutually exclusive MDM2, MDM4, and CDK6 amplifications and share a common DNA methylation signature. Mod Pathol. (2021) 34:2122–9. doi: 10.1038/s41379-021-00874-y. PMID: 34312479 PMC8592836

[21] Park C Kim R Bae JM Lee T Song S Kwak Y . Genomic profiling of intimal sarcoma reveals molecular subtypes with distinct tumor microenvironments and therapeutic implications. ESMO Open. (2025) 10:104097. doi: 10.1016/j.esmoop.2024.104097. PMID: 39778225 PMC11758979

