# Supplementary material for: Surgery-enabled precision oncology in an MSI-High pulmonary artery sarcoma with Lynch syndrome: a case report
Source: Front Oncol. 2026 Apr 30;16:1822606. doi: 10.3389/fonc.2026.1822606 (PMC13171380; doi:10.3389/fonc.2026.1822606)
Supplement: Supplementary file 1 [file DataSheet1.docx]

Supplementary Table 1

Genetic abnormalities possibly associated with the tumor’s pathogenesis were identified, including:

• AKT3 amplification (7 copies)

• ARAF amplification (10 copies)

• ATRX frameshift mutation (D1940fs*15)

• BCOR large-scale mutation

• CIC missense mutation (R2221H)

• EED nonsense mutation (R414*)

• FLCN frameshift mutation (H429fs*27)

• HDAC1 missense mutation (R431C)

• INPP4B frameshift mutation (L93fs*1)

• MET amplification (29 copies)

• MLL2 frameshift mutation (P443fs*487)

• MSH3 frameshift mutation (N385fs*19)

• QKI missense mutation (A313V)

• STD2 frameshift mutation (T305fs*35)

• TP53 frameshift mutation (S362fs*8, VAF 21.76%)

• TP53 nonsense mutation (R342*, VAF 87.94%)

• TSC2 frameshift mutation (G654fs*45)
