# Supplementary figures and images for: Surgery-enabled precision oncology in an MSI-High pulmonary artery sarcoma with Lynch syndrome: a case report
Source: Front Oncol. 2026 Apr 30;16:1822606. doi: 10.3389/fonc.2026.1822606 (PMC13171380; doi:10.3389/fonc.2026.1822606)

I

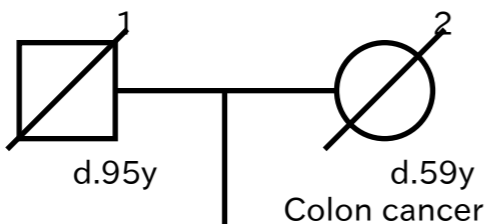

II

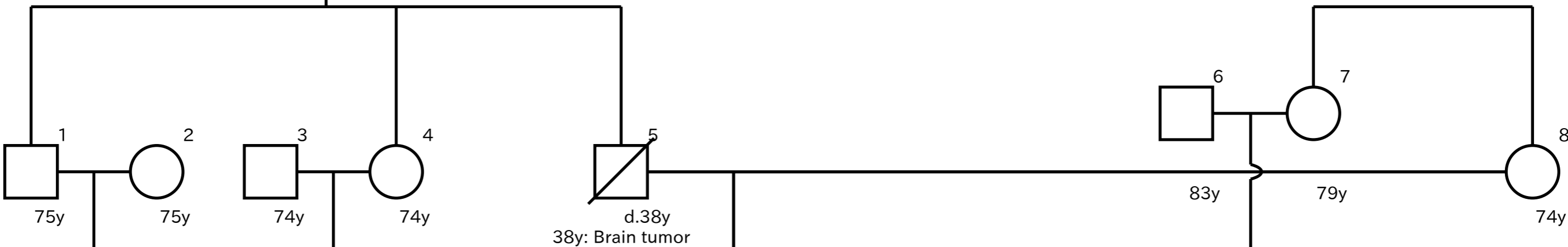

III

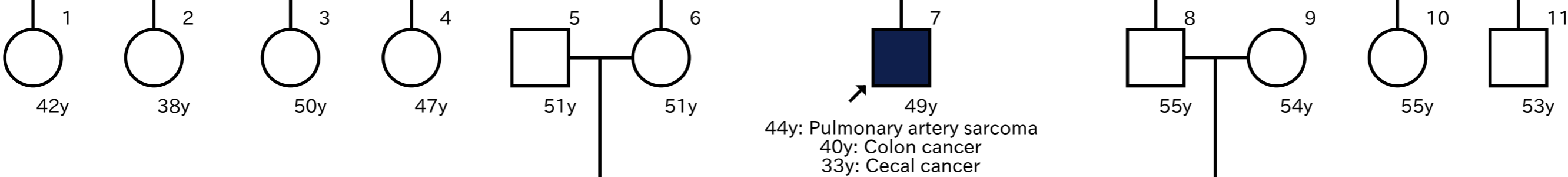

IV

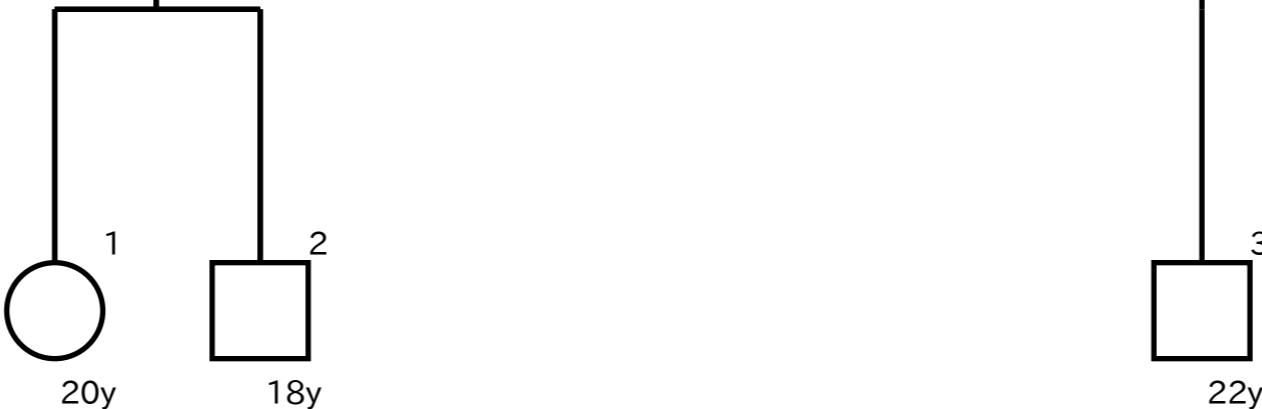

Supplement: Supplementary file 2 [file DataSheet2.pdf]

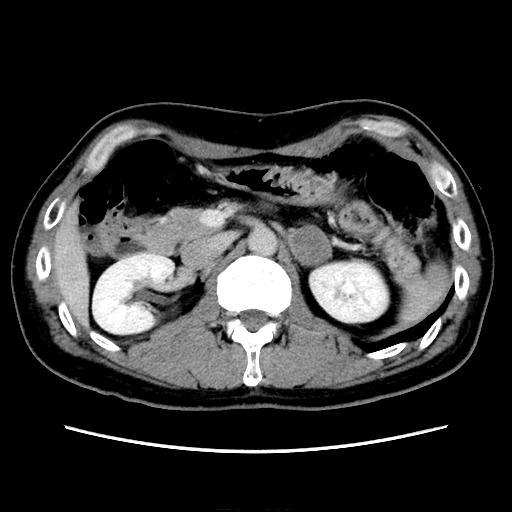

Supplement: Supplementary file 3 [file Image1.tif]
